# Prevalence of Mental Illness, Cognitive Disability, and Their Overlap among the Homeless in Nagoya, Japan

**DOI:** 10.1371/journal.pone.0138052

**Published:** 2015-09-17

**Authors:** Akihiro Nishio, Mayumi Yamamoto, Ryo Horita, Tadahiro Sado, Hirofumi Ueki, Takahiro Watanabe, Ryosuke Uehara, Toshiki Shioiri

**Affiliations:** 1 Health Administration Center, Gifu University, 1–1 Yanagido, Gifu, 501–1193, Japan; 2 Department of Psychopathology, Division of Neuroscience, Graduate School of Medicine, Gifu University, 1–1 Yanagido, Gifu, 501–1193, Japan; 3 United Graduate School of Drug Discovery and Medical Information Sciences, Gifu University, 1–1 Yanagido, Gifu, 501–1193, Japan; 4 Faculty of Health Promotional Sciences, Tokoha University, 1230 Kitaku, Miyakoda, Hamamatsu, 431–2102, Japan; 5 Midori Hospital, 1-14-24 Kitayama, Gifu, 501–3113, Japan; 6 Yoshida Hospital, 1-7-1 Saidaiji Akoda, Nara, 631–0818, Japan; Institute of Psychiatry, UNITED KINGDOM

## Abstract

**Background:**

While the prevalence of mental illness or cognitive disability is higher among homeless people than the general population in Western countries, few studies have investigated its prevalence in Japan or other Asian countries. The present study conducted a survey to comprehensively assess prevalence of mental illness, cognitive disability, and their overlap among homeless individuals living in Nagoya, Japan.

**Methods:**

Participants were 114 homeless individuals. Mental illness was diagnosed based on semi-structured interviews conducted by psychiatrists. The Wechsler Adult Intelligence Scale-III (WAIS-III, simplified version) was used to diagnose intellectual/ cognitive disability.

**Results:**

Among all participants, 42.1% (95% CI 33.4–51.3%) were diagnosed with a mental illness: 4.4% (95% CI 1.9–9.9%) with schizophrenia or other psychotic disorder, 17.5% (95% CI 11.6–25.6%) with a mood disorder, 2.6% (95% CI 0.9–7.5%) with an anxiety disorder, 14.0% (95% CI 8.8–21.6%) with a substance-related disorder, and 3.5% (95% CI 1.4–8.8%) with a personality disorder. Additionally, 34.2% (95% CI 26.1–43.3%) demonstrated cognitive disability: 20.2% (95% CI 13.8–28.5%) had mild and 14.0% (95% CI 8.8–21.6%) had moderate or severe disability. The percent overlap between mental illness and cognitive disability was 15.8% (95% CI 10.2–23.6%). Only 39.5% (95% CI 26.1–43.3%) of the participants were considered to have no psychological or cognitive dysfunction. Participants were divided into four groups based on the presence or absence of mental illness and/or cognitive disability. Only individuals with a cognitive disability reported a significant tendency toward not wanting to leave their homeless life.

**Conclusion:**

This is the first report showing that the prevalence of mental illness and/or cognitive disability among homeless individuals is much higher than in the general Japanese population. Appropriate support strategies should be devised and executed based on the specificities of an individual’s psychological and cognitive condition.

## Introduction

The prevalence of mental illness and/or intellectual/cognitive disability is higher among homeless individuals compared to the general population in Western countries [1–5]. Fazel et al. reported the prevalence of psychotic illness, major depression, alcohol and drug dependence, and personality disorders based on a systematic review and meta-regression analysis that included 29 eligible surveys with 5,684 homeless individuals [6]. According to their report, the prevalence of psychotic illness among the homeless in Western countries was 12.7% (95% confidence interval [CI] 10.2–15.2%); major depression was 11.4% (95% CI 8.4–14.4%); personality disorders was 23.1% (95% CI 15.5–30.8%); alcohol dependence was 37.9% (95% CI 27.8–48.0%); and drug dependence was 24.4% (95% CI 27.8–48.0%) between 1966–2007. Although there are several reports linking mental illness to homelessness, few studies have examined intellectual/cognitive disabilities among Western homeless individuals. For example, only four previous studies measured IQ scores in adult homeless samples within the past two decades (1995–2014). Reports from Canada, the Netherlands, the UK, and US, have demonstrated that the percentage of intellectual/cognitive disability among homeless samples ranges from 6 to 34% [7–10]. Compared to the prevalence of intellectual/cognitive disability in the general population of Western countries (10.37 per 1,000 individuals, reported by Pallab [11]), the prevalence of intellectual/cognitive disability among homeless individuals was much higher in the four aforementioned studies. Conversely, even fewer studies have assessed prevalence rates of mental illness among homeless individuals in Japan or anywhere else in Asia. Only the “Ikebukuro Station Study” (2009) demonstrated that 62.5% of homeless individuals in Tokyo had a diagnosable mental illness[12]. To our knowledge, no academic study has previously examined the prevalence of intellectual/cognitive disability among Japanese or Asian homeless samples. Furthermore, the relationship between mental illness and intellectual/cognitive disability has not been addressed in these samples, even though mental illness and intellectual/cognitive disability overlap frequently in the general population. When investigating the prevalence of cognitive disabilities among homeless individuals, etiology of the disability should be distinguished between congenital issues, the influences of general aging, and influences related to a mental illness. These distinctions are necessary in order to determine an appropriate support system that caters to a disability’s source. Therefore, the present study included mental illness diagnostics and assessments of congenital intellectual disability or acquired cognitive disability to better demonstrate the prevalence and overlap between these two concepts in Nagoya, Japan.

Nagoya is the fourth largest urban city in Japan, with approximately 2.3 million people. According to a 2012 report conducted by the Nagoya local government, there were 347 homeless individuals in Nagoya city [13]. The total number of homeless individuals in Japan is estimated to be 9576 as of 2012 [13]. Thus approximately 3.6% of the Japanese homeless population live in Nagoya. However, these numbers include individuals living outdoors, not who living in a temporary residence, netcafé, or other subsidized housing. More than 90% of the homeless are men. Nagoya has two large non-governmental organizations (NGO) that support homeless individuals; the Sasashima Support Center (SSC), which provides free physical examinations and social welfare consultation and-Sasashima Kyoseikai (SK),- which provides food services and welfare consultation in Nagoya. These two NGOs are partly financed by Nagoya city. There are also additional small NGOs for the homeless in Nagoya. However their contributions are limited. Our survey was proposed by the SSC and conducted with support of the SSC and SK as well as other NGOs with awareness that many individuals were not able to escape from their situation and seemed to be hampered from a life on the street because of mental or cognitive problems. Thus, more precise knowledge regarding the relationship between mental illness and cognitive disabilities may help facilitate effective support systems. We previously reported that the prevalence of mental illness, cognitive disability, and the overlap between the two was, 61%, 39%, and 28%, respectively, based on a survey of 18 homeless individuals in, Nagoya, Japan [14]. However, given the limited sample size in that previous study, the present study increased the target group and revised the requisite survey methods.

## Methods

### Design and participants

Informed consent was conducted on 114 homeless adults on November 2, 2014, in Nagoya, Japan. The definition of homeless was taken from the Department of Health and Human Services in the US; Someone who is “homeless” is “an individual who lacks housing without regard to whether the individual is a member of a family, including an individual whose primary residence during the night is a supervised public or private facility (e.g., shelters) that provides temporary living accommodations, and an individual who is a resident in transitional housing [15]. In the present study, homeless individuals were recruited in cooperation with the Sasashima Support Center. The survey was advertised with compensation coming in the form of a prepaid card in the meal service place.

### Ethics statement

The Ethical Review Committee, Graduate School of Medicine, Gifu University, approved this research protocol on August 6, 2014 (approval No. 26–133). All participants were given detailed face-to-face explanations regarding the protocol by medical professionals and provided written informed consent. This informed consent form was written in manner that made it easy to read (at reading level commensurate with IQ score below 69). Based on interviews and medical records, participants who required medical care or welfare services were referred to the appropriate medical institutions at the time of the survey.

## Measurements

In rented, quiet, and separate meeting rooms within an office building located near the Nagoya station, the psychiatric diagnostic interview, the Wechsler Adult Intelligence Scale version III (WAIS-III) [16], and a semi-structured interview and hearing examination were conducted by accredited psychiatrists, clinical psychologists, and other medical professionals. Medical professionals assessed participant age, length of homeless life, age when street life was initiated, education level, history of drinking/ smoking/ gambling, welfare notebook and pension status, history of receiving welfare, and experience with psychiatric consultation. The psychiatrists interviewed participants through semi-structured interviews using the Mini-International Neuropsychiatric Interview based on diagnostic criteria from the Diagnostic and Statistical Manual of Mental Disorders, Fourth Edition, Text Revision (DSM-IV-TR). Clinical psychologists assessed current and peak intellectual capacity for each participant using the WAIS-III (simplified version); psychiatrists administered the Japanese Adult Reading Test (JART). Although there are several approaches to the simplified version of the WAIS-III, we used Dairoku et al.’s method [17], which carried out 4 subtests from 13 possible; Picture Completion, Digit Symbol-Coding, Digit Span, and Information. The four total scores were doubled and 20 points was added. This method produced a reliability coefficient = 0.93, and a coefficient of validity = 0.88 [16]. Scores on the WAIS-III and JART were compared within each individual. The JART is a test measuring intellectual capacity, which was developed based on the National Adult Reading Test (NART). The JART measures intellectual capacity under the assumption that reading Kanji (Japanese version of Chinese characters) is highly correlated with mental capacity. Since it is rare for people to lose their ability to read Kanji, despite a decrease in cognitive functioning due to aging or the effects of psychosis, administering JART is suitable as it is less susceptible to changes [18, 19]. JART specificity was used to estimate each participant's peak intellectual capacity. Therefore, we were able to determine whether a participant’s diminished mental capacity was congenital or acquired by measuring and comparing the WAIS-III and JART scores. Although low intellectual capacity is caused by congenital intellectual disability and acquired cognitive disability, we defined both as cognitive disability in this paper. Although some previous reports excluded acquired cognitive disability after 18 years old by taking history, we did not exclude cognitive disability acquired. The reason for this was that we considered acquired cognitive disability to be important and we could distinguish between congenital and acquired cognitive disability by comparing WAIS-III and JART scores. We divided the participants into groups by their WAIS IQ; 55–69, 40–54, below 40 as mild cognitive disability, moderate cognitive disability, and severe cognitive disability, respectively [20].

The 114 participants were divided into four groups according to the following: A, absent a mental/cognitive diagnosis; B, only a cognitive disability; C, only a mental illness; and D, both a cognitive disability and mental illness. Between each of the four groups, the following items were compared: mean age, gender, mean current homeless life length, residence, habitual smoking percentage, habitual drinking percentage. Participants responded to the following Likert scale item [21] (“strongly” to “not at all”); “Would you like to leave your homeless life?” This question was based on our experience that some homeless individuals express not wanting to leave their homeless life. However, it seemed these responses were not necessarily true and we thought we could evaluate their motivation for leaving their homeless life if the question was posed on a Likert scale. Definitions for “habitual smoking” and “habitual drinking” were based on the Japanese national health/nutrition survey criteria [22]. Smoking sometimes or every day during the past month, and drinking more than 180 ml of Japanese sake (almost equal in alcohol to a glass of wine) more than 3 times per week, were determined has habitual smoking and drinking, respectively. A gambler was defined as participants who were engaged in gambling activity at the time of the survey.

### Statistical analyses

Statistical analyses were performed using JMP® ver. 10.0.2 (SAS Institute, Tokyo, Japan). Analysis of variance (ANOVA) was used to compare age and period of homeless life among the A-D groups. Chi-square tests were used to compare gender, residence, history of smoking, history of drinking, and history of gambling in the A versus B, C, and D groups.

## Results

### Participant characteristics

Socio-demographic variables from the 114 participants (including 106 men and 8 women) are shown in Table 1. All participants were of Japanese descent and nationality. Participants' ages ranged from 20 to 78 years old, with a mean age of 54.0 ± 12.6 (mean ± SD). Seventy-two participants lived on the street, and 35 lived in temporary residences. Seven lived elsewhere or gave no response. Duration of current homeless life varied, ranging from less than one year to 20 years, with an average of 3.5 ± 4.4 years. Only 7 participants were taking psychiatric medication or receiving mental health consultation at the time of the interview; 21 participants had a history of psychiatric medication use or consultation. Thirteen participants received an elder adult pension, disability, or other pensions. Seventeen participants imbibed in alcohol (more than 11 Japanese wine units per week). Eighty-one participants were regular smokers. Finally, 38 participants were regular gamblers, and another 50 had previous gambling experience.

**Table 1:** Participant demographic characteristics.

Gender	Male	Female						
	106	8						
Age (years old)	20–29	30–39	40–49	50–59	60–69	Over 70		
	5	13	22	31	33	10		
Residence	Street	Temporary residence	Other	No response				
	72	35	4	3				
Duration of homeless life	1 and under	Over1 to 2	Over 2 to 3	Over 3 to 4	Over 4 to5	Over 5 to 9	Over 10	
	66	9	8	5	6	5	15	
Psychiatric consultation (present)	Yes	No						
	7	107						
Psychiatric consultation (past)	Yes	No						
	21	93						
Pension	No pension	Basic pension	Employees' pension	Disability pension	Other	Unknown		
	101	4	6	1	1	1		
Alcohol consumption (Number of 200ml wine bottle/week)	No drinking	1	2	3	4–5	6–10	11–20	More
	69	3	10	2	3	10	8	9
Smoking (Number of cigarette/day)	No smoking	1–10	11–20	21–30	More			
	33	29	44	3	5			
Ganbling	Sometimes	Not currently	Never					
	38	50	26					

### Mental illness and cognitive disability diagnoses

A mental illness diagnosis by certified psychiatrists, and cognitive disability measured with WAIS-III scores are shown in Table 2. Of the participants, 42.1% (95% CI 33.4–51.3%) were diagnosed with a mental illness as follows: 4.4% (95% CI 1.9–9.9%) with schizophrenia or other psychotic disorders, 17.5% (95% CI 11.6–25.6%) with a mood disorder, 2.6% (95% CI 0.9–7.5%) with an anxiety disorder, 3.5% (95% CI 1.4–8.8%) with a personality disorder, and 14.3% (95% CI 8.8–21.6%) with a substance-related disorder specific to alcohol dependence/abuse. No participant was diagnosed with illegal drug dependence/abuse.

**Table 2:** Relationships between diagnosed mental illness and cognitive disabilities.

	Normal intelligence	Mild intellectual disability	Moderate-severe Intellectual disability	Total
No mental illness	45 (39.5%)	14 (12.2%)	7 (6.1%)	66 (57.9%)
Having mental illness	30 (26.3%)	9 (7.9%)	9 (7.9%)	48 (42.1%)
1.Schizophrenia or other psychotic disorder	3	1	1	5 (4.4%)
2. Mood disorder	10	5	5	20 (17.5%)
3. Anxiety disorder	2	0	1	3 (2.6%)
4. Substance-related disorder	13	1	2	16 (14.0%)
5. Personality disorder	2	2	0	4 (3.5%)
Total	75 (65.8%)	23 (20.2%)	16 (14.0%)	114 (100%)

Mean IQ was 79.2 ± 21.0 (95% CI 83.1–75.3), excluding one participant who had an IQ too low to be measured with the WAIS–III. 34.2% (95% CI 26.1–43.3%) of participants were diagnosed with a cognitive disability, including 20.2% (95% CI 13.8–28.5%) with a mild cognitive disability and 14.0% (95% CI 8.8–21.6%) with a moderate/severe cognitive disability. We also investigated the overlap in mental illness and cognitive disability diagnoses. 15.8% (95% CI 10.2–23.6%) had both a diagnosed mental illness and cognitive disability. Only 39.5% (95% CI 26.1–43.3%) were considered to have neither an cognitive disability nor a diagnosed mental illness.

**Fig 1:**
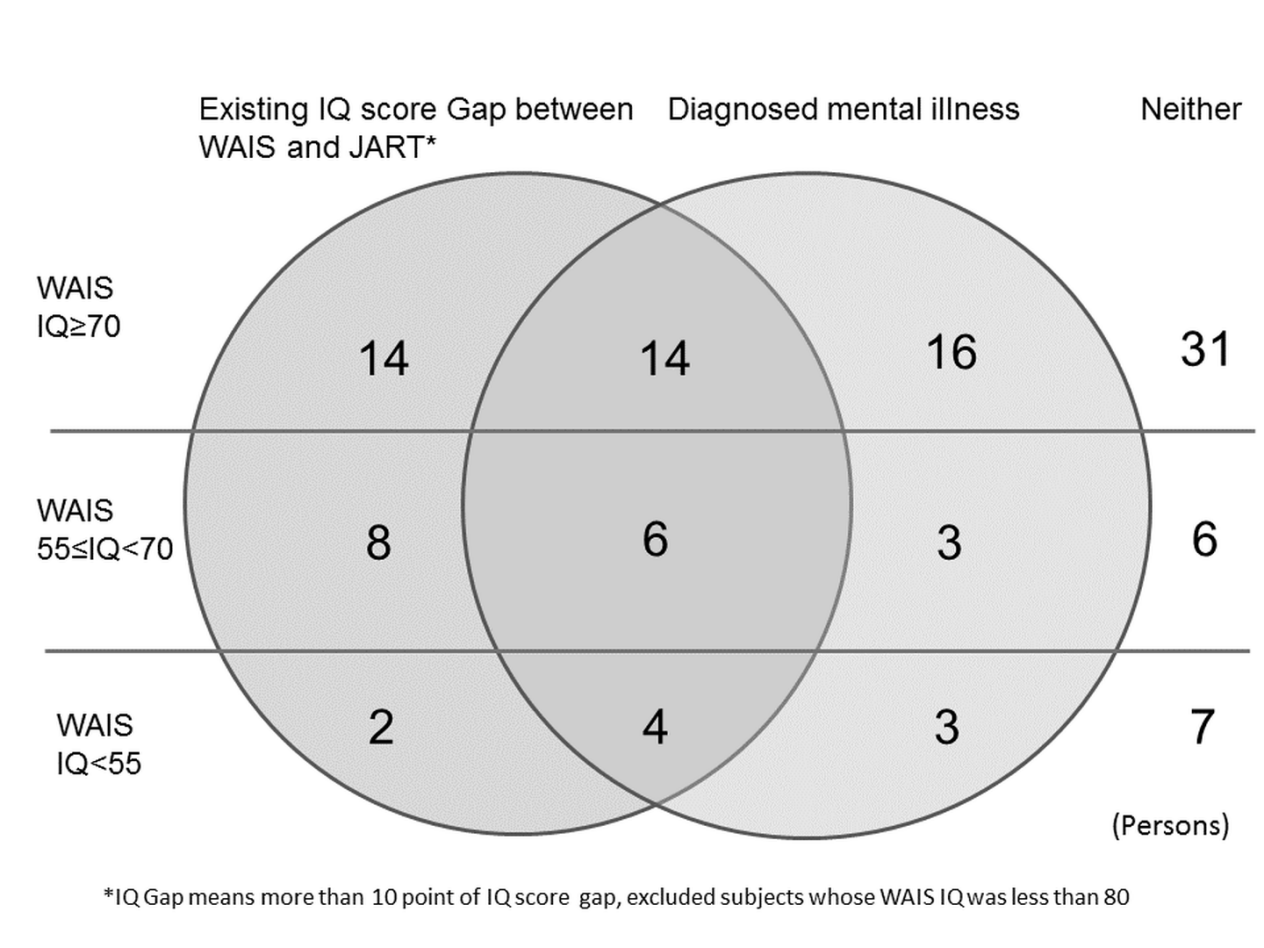
Relationship between IQ score gap and mental illness.

From comparisons between the WAIS-III and JART, 64 participants showed decrements that were larger than a normal range of dissociation (11 points). Since the JART cannot measure IQ scores lower than 69, participants with low IQ values due to developmental issues likely would show a greater dissociation between IQ scores and the JART. Thus, we excluded participants with a JART-estimated IQ score that was within the spectrum of cognitive disability or borderline (IQ < 80). Forty-eight participants met this criterion; 24 that had a diagnosed mental illness (Fig 1), 14 with no cognitive disability, 6 with mild cognitive disability, 4 with moderate-severe cognitive disability. Among those diagnosed with mental illness, 2 were diagnosed with schizophrenia or other psychotic disorder, 10 with a mood disorder, 2 with an anxiety disorder, 9 with a substance-related disorder, and 1 with a personality disorder. It was suggested that participants’ mental illness might have impaired cognitive ability in some cases. Twenty participants were estimated to have acquired their cognitive disability because of mental illness or other reasons, even though they likely had normal intellectual functioning at previous ages. Consequently, the estimated number of individuals with an intellectual disability, excluding those with intellectual decline due to aging or mental illness, was 19 (16.7%). Approximately half of the participants who had a cognitive disability developed their dysfunction over time.

Participants were divided into 4 groups according to the presence/absence of an intellectual disability or mental illness, and were analyzed (Table 3). There were no significant differences between groups in terms of mean age, gender, residence, time since becoming homeless, and habitual smoking, drinking, and gambling rates (ANOVA).

**Table 3:** Socio-demographic variables across the four participant groups.

	Normal	Intellectual Disability	Mental Illness	Intellectual Disability + Mental Illness	Total
Mean age ±SD (y.o.)	55.0 ±11.7	51.3 ± 15.2	56.5 ± 10.9	50.1 ±13.9	54.0 ± 12.6
Gender male (%)	93.3	90.5	100.0	83.3	93.0
Residence % street living	66.7	65.0	69.0	68.8	63.1
Period of homeless life (mean ± SD)(years)	3.6 ± 4.7	4.0 ± 5.4	3.1 ± 3.8	2.9 ± 3.3	3.5 ± 4.4
Habitual smoking rate (%)	68.9	81.0	66.7	72.2	71.1
Habitual drinking rate (%)	22.2	23.8	43.3	22.2	27.2
Gambling rate (%)	37.8	38.1	33.3	16.7	33.3

**Fig 2:**
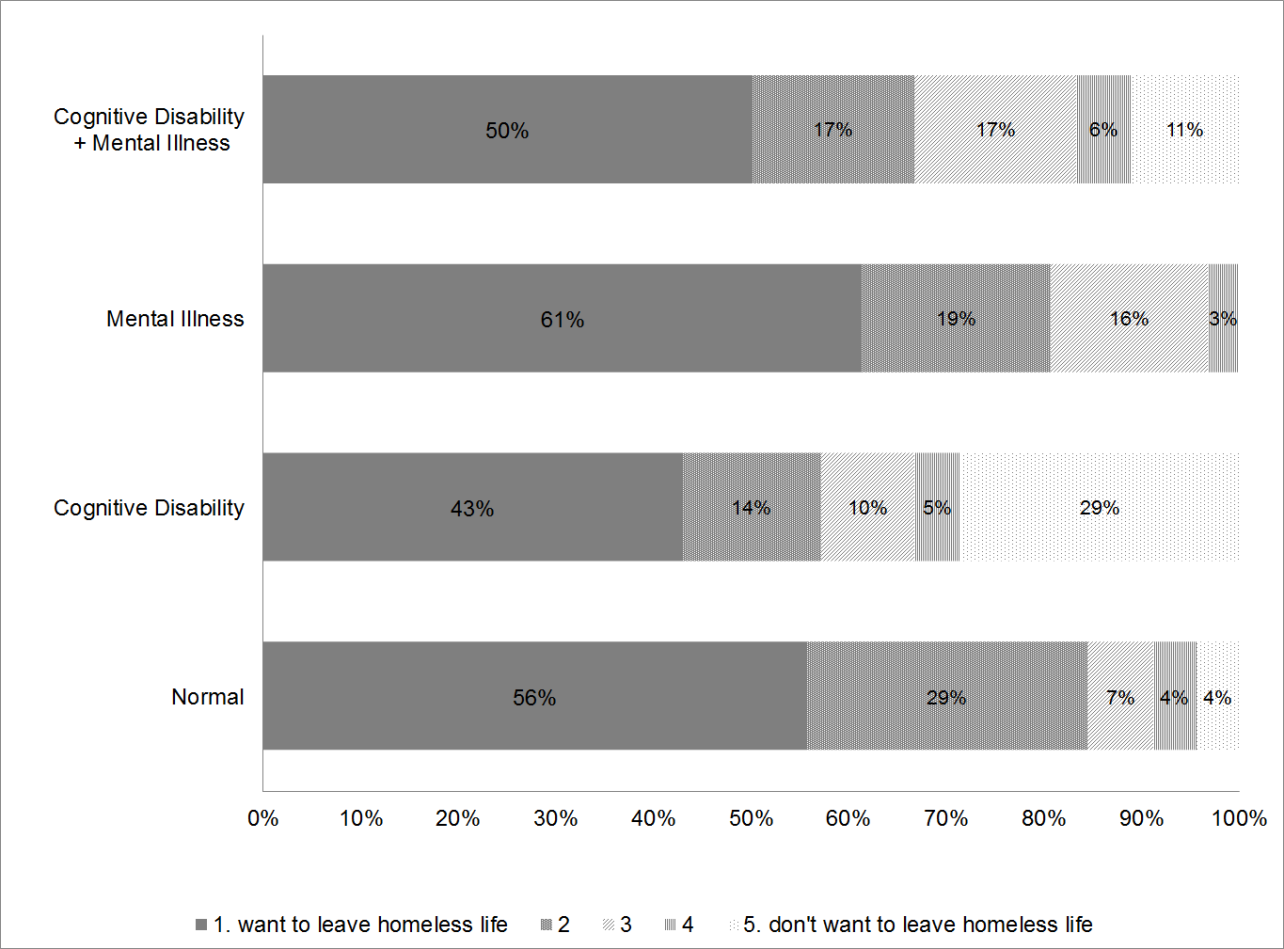
Responses to the question, “Would you like to leave your homeless life?” across the four participant groups.

Participants’ responses to the question are shown in Fig 2. In the cognitive disability group, 29% of participants stated, “I don’t want to leave my homeless life at all,” whereas only 4% and 0% of participants in the mental illness group and non-mental illness/cognitive disability group, respectively, wished to stay homeless. These percentages were significantly different (Chi-squared test, p = .0029). The following breakdown describes participants who answered a 3, 4, or 5 on this item: 44% of individuals with only a cognitive disability and 15% without a cognitive disability or diagnosed mental illness.

## Discussion

The current study had the following aims: (1) investigate the prevalence of cognitive disability, mental illness, and their overlap in a Japanese homeless sample and; (2) clarify potential differences in needed support among these four sample groups.

We have already reported the prevalence of cognitive disability and mental illness in a small sample of Japanese homeless residents [13]. The present study is an extension of that previous report that includes a larger sample. The present survey revealed a prevalence rate of homeless adults with mental illness at 42.1%. The “Ikebukuro Station Survey” investigated 80 homeless individuals living within a 1-km radius of the Ikebukuro station in Tokyo, observing a mental illness prevalence of 62.5%, including 41.3, 15.0, and 15.0% with depression, alcohol dependence, and psychotic disorders, respectively [12]. While the Ikebukuro survey revealed a higher mental illness prevalence rate, the percentage of alcohol dependence/abuse was almost identical between our study and theirs. Since the Ikebukuro Station Study was conducted by an NGO, which specializes in mental health support for homeless individuals, the target samples between the two studies are a little different. For instance, our current survey was conducted in Nagoya, where several NGOs are in cooperation. This allowed us to target a wide range of individuals, perhaps more reflective of the general mental health status of homeless Japanese residents.

Compared to prevalence rates in Western countries reported by Fazel and colleagues [6], depression prevalence was higher, while psychotic disease, substance related disorder, and personality disorder prevalence was lower in the present sample. According to mental illness prevalence reports in Japan conducted by the WHO, the average rate of mental illness was 8.8% (6.4–11.2, 95%CI), including 5.3% for anxiety, 3.1% for mood disorders, 1.0% for impulse-control disorders, and 1.7% for substance dependence [23]. These data did not include rates for schizophrenia and psychosis. Nakane et al. reported that schizophrenia prevalence ranged from 0.21% to 1.79% in the general Japanese population [24]. Taking these aforementioned prevalence rates into consideration, mental illness prevalence in the general Japanese population would be roughly 10%. Thus, the current mental illness rate observed among homeless individuals in Nagoya (42.1%) is much higher than the general population.

The current study also measured intellectual capacities among this homeless sample; 34.2% presented with a cognitive disability, including 20.2% with mild and 14.0% with moderate to severe disability. Okuda [25] observed that 56 of 164 participants (34.2%) had a cognitive disability (IQ < 70) among Japanese homeless individuals based on a field study. While Okuda’s survey was conducted in the field, disability rates between Okuda’s and the present study are identical. Since the prevalence of whole cognitive disability (IQ < 70) in the general population was statistically estimated at 2.5%, prevalence rates observed in the current study are rather high.

Over the past 20 years (1994–2013), only four previous studies have reported IQ scores obtained from adult homeless samples within Western countries. One was performed with a very young homeless sample between the ages of 16 and 21 years old in the US, observing that 6% of the sample had IQ scores within or at the border of the cognitive disability range (IQ < 80) [7]. The second study assessed 50 homeless individuals with a mean age of 33.6 ± 10.5 in the UK, reporting that 10% showed IQ scores within an cognitive disability range (IQ < 70) [8]. The third study reported that 34% of homeless individuals, ranging in age from 24 to 68 years old (and who were receiving public health services) in Canada, presented with intellectual/cognitive disabilities [9]. Although the 34% rate from a Canadian sample was quite different from those observed in the US and UK, it should be noted that the Canadian study was based on both historic and current disability profiles, not based on standardized tests (e.g., the WAIS). Furthermore, that study was limited to individuals receiving public health services. The fourth study was from the Netherlands, reporting a 29.5% cognitive disability rate for a sample with a mean age of 39.9 ± 13.0 [10]. Although the cognitive disability prevalence reported in the present study (34.2%) was higher than that in these aforementioned studies, the rate observed in the present study should be considered in light of potential declines resulting from normative age effects and concomitant mental illness. The present sample was substantially older compared to the four aforementioned studies. From a 2012 fact-finding report referred to as the “Homeless Person’s Life”, conducted in Nagoya, mean respondent age was 58.8 years [26]. The mean age of the current sample was 54.0 ± 12.6 years old, accurately reflecting the average age of Japanese homeless residents. To distinguish declines in intellectual capacity resulting from general aging or mental illness, we compared WAIS-III and JART scores. This comparison revealed that 50% of participants with a cognitive disability did not come by their dysfunction congenitally; rather, it likely developed over time. Thus, higher rates of cognitive disability among the homeless in Japan may reflect several non-congenital factors such as age-related decline and dysfunction due to mental illness.

The present study also investigated the overlap between mental illness and cognitive disability among homeless individuals. 15.8% of participants were considered to have both a mental illness and cognitive disability. Only 39.4% of participants were absent either a mental illness or cognitive disability. In other words, 60.6% of the present sample had at least one or the other. This high proportion of mental and cognitive difficulties should make it apparent that this segment of the population is in need of intervention. Thus, additional research should provide insight as to the support needed for homeless individuals with mental illness and/or cognitive disability.

The present study also assessed rates of habitual smoking, drinking, and gambling. Results showed that rates of habitual smoking and gambling were rather high relative to participants’ rather low incomes. The habitual smoking rate was 71.1% in the present sample, which is much higher than the rate among Japanese men in the general population (34.1%) compiled in 2012 [22]. High smoking rates among homeless residents are comparable to what is observed in the US [27, 28]. Conversely, the rate of habitual drinking (27.2%) was lower among the homeless compared to the general male Japanese population (34.0%) observed in 2012 [22]. This was in spite of 16 individuals within our sample (14.0%) diagnosed with alcohol dependency/abuse. It is undetermined whether the gambling rate observed in the present survey was high compared to the general population, or whether these addiction rates are problematic. This is because problematic gambling was difficult to define by solely using the present survey. Nevertheless, rates of habitual smoking and gambling could be seen as detrimental given the low-income levels participants had for supporting such habits. It is possible that such habits helped facilitate their homelessness or kept some individuals from moving out of their current circumstances. Thus, it should be noted that one issue related to individuals’ difficulty in leaving their homeless life could be their addictive tendencies; thus, addiction support would be a necessary point for intervention.

The present sample was divided into four groups based on the presence or absence of mental illness/cognitive disability. There were no significant differences between groups on several socio-demographic factors, as well as habitual smoking, drinking, and gambling rates. Answers to the question, “Would you like to leave your homeless life?” showed that participants with a cognitive disability were less likely to answer this question in the affirmative. This finding suggests that such individuals may refuse social support and receipt of services due to their cognitive disability. Thus, one’s refusal to eschew their homeless life might not be fully willful; it might be due to an awareness of difficulties related to social adaptation. Thus, homeless individuals with cognitive disabilities should be supported by those who specialize in dealing with social adaptation issues as a way to allay these concerns.

## Conclusion

This is the first study to examine the prevalence of mental illness, cognitive disability, and the overlap between the two in a Japanese homeless sample. Approximately 60% of the individuals presented with a mental illness, a cognitive disability, or both; this rate is much higher than what is observed in the general Japanese population. To better maximize support systems for the homeless in Japan, an understanding of mental and psychological issues, beyond basic economic concerns, is tantamount to enhancing social welfare within this population.
